# Ethnobotanical survey of medicinal plant species used by communities around Mabira Central Forest Reserve, Uganda

**DOI:** 10.1186/s13002-015-0077-4

**Published:** 2016-01-13

**Authors:** Patience Tugume, Esezah K. Kakudidi, Mukadasi Buyinza, Justine Namaalwa, Maud Kamatenesi, Patrick Mucunguzi, James Kalema

**Affiliations:** Department of Biological Sciences, College of Natural Sciences, Makerere University, P.O Box 7062, Kampala, Uganda; College of Agriculture and Environmental Sciences, Makerere University, P.O Box 7062, Kampala, Uganda; Bishop Stuart University, P.O Box 9, Mbarara, Uganda

**Keywords:** Ethnobotanical, Medicinal plants, Mabira CFR, Fidelity level, Health conditions

## Abstract

**Background:**

An ethnobotanical study of medicinal plants was carried out in 14 villages adjacent to Mabira Central Forest Reserve (CFR) in Central Uganda between August 2013 and March 2014.

**Methods:**

Information was obtained through interviews using semi- structured questionnaires. Field excursions with traditional healers and herbal medicine collectors were carried out. Descriptive statistics were used to present the data. Fidelity ratios and Informant consensus agreements were calculated.

**Results:**

A total of 190 plant species in 61 families and 152 genera were reported in the treatment of various health conditions. Family Fabaceae was dominant representing 14 % of the plant species documented. *Vernonia amygdalina* was the preferred species for treating malaria. Leaves (68 %) were the most frequently used parts in preparing herbal remedies. Decoctions (29 %) and oral route (53 %) of administration were commonly used method of herbal medicine preparation and administration respectively. Fifty-eight health conditions grouped in 25 categories were treated using medicinal plants. Informant consensus agreement was highest for blood system disorders (0.9) that included anaemia, hypertension and blood cleansing indicating homogeneity of informant’s knowledge about remedies used. *Vernonia amygdalina and Erythrina abyssinica* had 100 % fidelity level for treatment of malaria and vomiting respectively.

**Conclusion:**

The diversity of medicinal plant species used and the associated indigenous knowledge are of great value to the local community and their conservation and preservation is paramount. The therapeutic uses of the documented plants provides basic data for further research focused on pharmacological studies and conservation of the most important species.

## Background

The acceptance and use of herbal medicine is on the increase globally [1–3]. In Africa the situation is not different, over 80 % of the population particularly in the developing countries depends directly on plants for their primary healthcare requirements [4, 5]. In the East African region countries such as Burundi [6] and Tanzania [7] that neighbour Uganda, the population using traditional medicine is also well above 80 % particularly in the rural areas [6, 7]. Plants form an important part of health care especially for the rural poor in Uganda [8]. The Ugandan government has specifically up scaled the use of herbal medicine and is in the process of integrating it into the main health care system [9, 10]. The noted increased use of herbal medicine is as a result of the confirmed therapeutic evidence of the herbal remedies [11]. This has been enhanced by the consequences of limited access to modern health services in most developing countries including Uganda, high cost of modern medicine compared to the indigenous herbal medicines, wide socio-cultural acceptance of traditional medicine and the belief that natural products pose no risk [3, 4, 12, 13].

The increased preference of herbal medicine has consequently propelled the search for pharmaceutical remedies against different ailments from plants [14]. The medicines are collected from the wild and this has negatively impacted on the plant resource due to unsustainable exploitation rates as well as the health of many people who cannot afford orthodox medicine [15–17]. This makes documentation, sustainable utilisation as well as conservation essential [3, 18]. The first step in conservation is to document material traditionally used to treat an ailment [15, 16]. Previous studies have identified and documented numerous medicinal plants for treatment of various diseases in Uganda [1, 19] however these have been targeting specific ailments and are not detailed in shared use. A larger number of medicinal plants and indigenous uses have not yet been documented. The rich history of African cultures and their innovative utilisation of plants as a source of remedies have been passed down through generations largely by oral tradition [20]. This knowledge is gradually being lost [21] as the custodians die before passing on information to the younger generations. Besides the gradual loss of ethnobotanical knowledge due to lack of documentation, overharvesting of medicinal materials from their natural habitat has been one of the major threats of traditional medicine. In order to conserve wild plant species, there is need for reliable data on their distribution and level of use [22].

The documentation of indigenous knowledge through ethnobotanical studies is important in conservation and utilization of biological resources [23]. The identification of local names, scientific names and indigenous uses of plants not only preserves indigenous knowledge but also facilitates future research on safety and efficacy of medicinal plants in treatment of various ailments [24]. It is against this background that utilization of medicinal plants as a source of primary health care by communities adjacent to Mabira CFR is documented. This will ensure that traditional knowledge about use of these plants is conserved. It will also facilitate the discovery of new sources of drugs and promote sustainable use of medicinal plant resources in Uganda. In addition conservation of medicinal plants will add value to the recreational environment as well as health improvement through sustained ecosystems. This study aimed at collecting data on plant species used to treat different health conditions by communities adjacent to Mabira CFR.

## Methods

### Study area

**Fig. 1:**
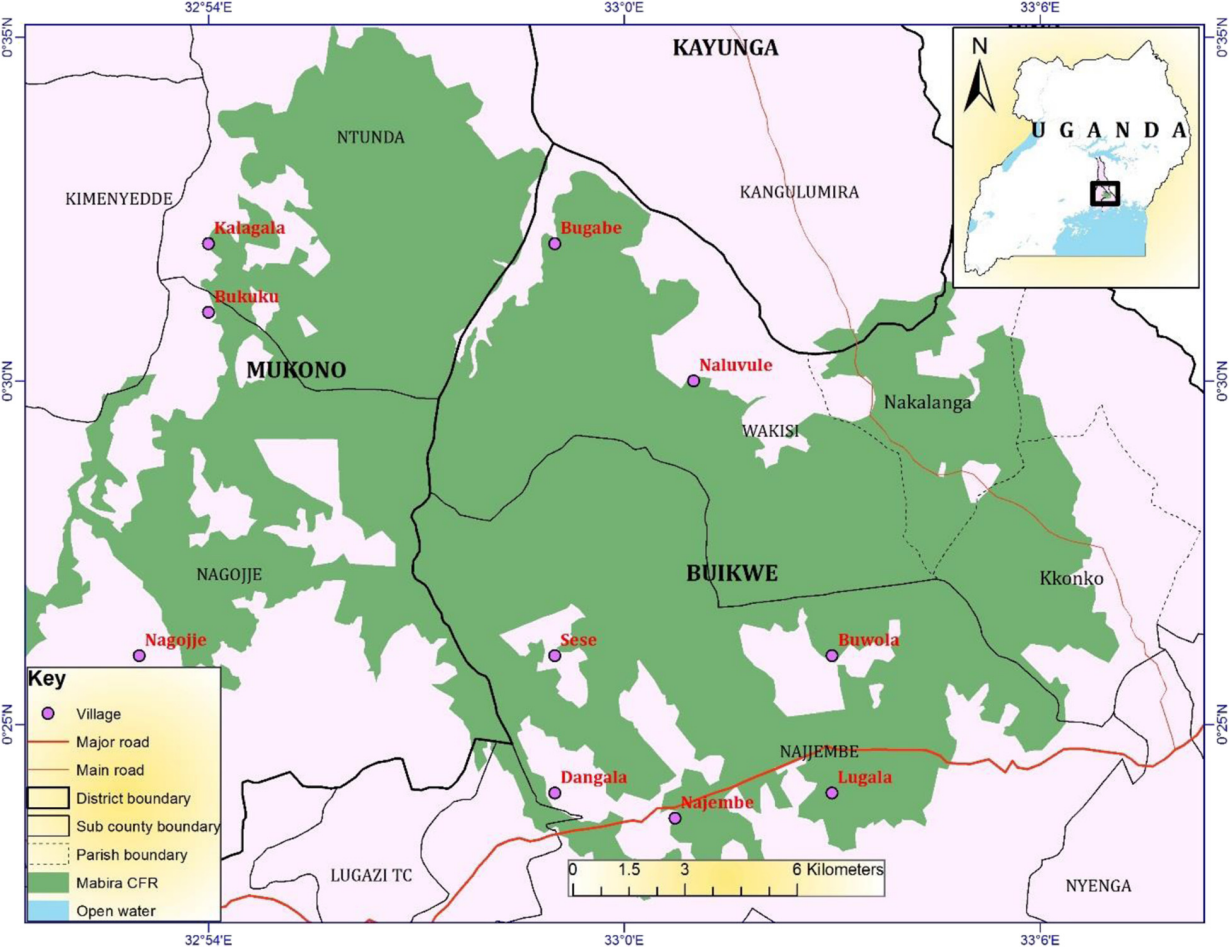
Map of Mabira CFR showing the study villages. The figure shows location of Mabira CFR in Uganda and specifically highlights the sites of villages where ethnobotanical surveys of medicinal plants were carried out. The map displays demarcations of the administrative boundaries showing the major road network and the main physical features in the study area

The study area covered human settlement areas around Mabira CFR some of which were enclaves and others adjacent to the forest. Mabira CFR is located 20 km north of Lake Victoria shoreline immediately to the west of Victoria Nile. The forest reserve lies partly in Buikwe, Mukono and Kayunga districts and occupies an area of 306 km^2^ with an altitudinal range of 1070 – 1340 m above sea level [25]. It is situated between latitude 0^o^ 22’ and 0^o^ 35’N and between longitude 32^o^ 56’and 33^o^ 02’E [26] (Fig. 1).

The forest reserve occupies gently undulating landscape characterised by numerous flat-topped hills (relics of the ancient African peneplain), and wide shallow valleys [27]. The topography is such that the land drains to the north, even though the reserve’s southern boundary lies only 13 km from the lakeshore. The underlying rocks are composed of micaceous schists and shales of the Buganda- Toro system with ridges of quartzite and amphibolite. The soils are generally ferralitic sandy clay loams, with black waterlogged clays in the valley bottoms. The climate is tropical with two rainfall peaks from April to May and October to November ranging between 1,250 – 1,400 mm per annum. Annual mean temperature range, minimum: 16–17 ° C, maximum: 28–29 ° C. The vegetation of Mabira CFR was classified as “medium altitude moist semi-deciduous [28].

Commercial use of the forest began when some parts were harvested in the early 1900’s and until 1988, intensive coffee/banana agricultural encroachment badly damaged parts of the forest. [27] About 21 % and 26 % of the reserve have been designated as strict nature reserve and buffer zone respectively and the forest in these areas is recovering following extensive plantings of native tree species.

The human population living in the forest enclaves was approximately 825,000 with a density of 200–230 people per Km^-2^ [29]. The local people are mainly of the Bantu ethnic group of the following tribes; Baganda, Banyarwanda, Basoga, Bagisu, Bakiga, Banyankole, Bagwere and Batoro.

The reserve has tea and sugarcane plantations around. Some local people reside in settlements for labourers on the tea and sugarcane estates [30]. The extent of growing cash crops other than tea and sugar cane is limited by scarcity of land. However locals are engaged in cultivation of food crops mainly for subsistence consumption like maize, beans, bananas, ground nuts, sweet potatoes and vegetables. Livestock rearing is limited to a few households.

### Ethical considerations

Ethical approval of the study was obtained from the Uganda National Council of Science and Technology (UNCST) under registration number SS 3368 after obtaining a research license from National forestry Authority (NFA).

### Data collection

This was a field survey targeting custodians of Traditional Medicine used in treatment of diseases. Verbal pre-informed consent was obtained from the participants before the interview. Interviews were conducted in Luganda the local language in the area using guided semi structured questionnaires and a research assistant that was conversant with the local language.

Collection of data on medicinal plants used to treat different ailments in the study area was according to a slight modification of Martin’s procedure [31]. Purposive sampling was used to identify 14 out of 27 villages that heavily depend on the forest for primary health care through a Rapid Rural Appraisal (RRA) with village leaders. Heavy dependence was defined by village council leaders’ local experience i.e. based on the number of individuals who depend wholly on herbal medicine for livelihoods. The study included villages within 1–5Km from the forest. This is because distance from the forest influence people’s use of forest products. Before entering each of the villages, permission was sought from local leaders after explaining the aim of the study who gave us the name of the first key informant while the rest of the respondents were selected by snow ball sampling technique. [32, 33] A total of 36 key informants were selected with at least two from each village and an additional eight knowledgeable herbalists recommended by the community members from Naluvule, Bukuku, Buwoola and Kalagala villages. The informants included primary collectors, vendors and traditional healers who are the custodians of indigenous knowledge on herbal medicines. Traditional healers are divided into two broad groups of herbalists who mainly use herbs while diviners also invoke ancestral spirits to guide them in their healing practice [34–36]. They provided information on plants and parts used, ailments treated, mode of preparation and administration, habit, source and availability of medicinal plants. Field excursions were conducted along forest trails taking traditional healers as guides and voucher specimens of cited medicinal plants were collected.

### Preference ranking

Preference ranking [31] of the 10 most available medicinal plant species and diseases commonly treated by each were shortlisted by the 12 key informants according to importance attached to the species as per frequency of use and effectiveness (number of days taken to healing in treating particular diseases successfully). The values assigned for each species across were summed up for all the informants to get an overall rank value. The species were then ranked in descending order with the species that had the highest total ranked first.

### Plant identification and processing of Voucher specimens

Plant identification was partly carried out in the field based on field manuals for plant identification [37, 38]. Voucher specimens were collected and later identified at Makerere University Herbarium. Correctness of scientific names of species were also checked according to Tropicos:http://www.tropicos.org database accessed on 12/05/2015.

### Data analysis

Descriptive statistics using frequencies and percentages were used to summarize data using Microsoft excel 2013. The ailments treated by the medicinal plants were classified into different categories [39].

### Informant consensus agreement

The informant consensus factor (F_ic_) was calculated to indicate the homogeny of information using the formula;

$$ \begin{array}{rcl}{\mathrm{F}}_{\mathrm{ic}}& =& \frac{{\mathrm{N}}_{\mathrm{ur}} - {\mathrm{N}}_{\mathrm{taxa}}}{{\mathrm{N}}_{\mathrm{ur}}-1}\\ {}\mathrm{Where}\ {\mathrm{N}}_{\mathrm{ur}}& =& \mathrm{Number}\ \mathrm{of}\ \mathrm{use}\ \mathrm{reports}\end{array} $$

N_taxa_ = Number of species in each use category. It estimates the relationship between the number of use reports (N_ur_) minus the number of taxa used (N_taxa_) and the number of use reports in each category minus one [40].

F_ic_ values are low if plants are chosen randomly or if informants do not exchange information about their use or disagree about the species used in treatment of an ailment category. The values are high (close to one) if the species are used by a large proportion of informants and there is a well-designed criterion in community or if information is exchanged between informants. Therefore the medicinal plants are presumed to be effective in treating a certain disease have higher F_ic_ values [41].

### Fidelity level (FL)

Fidelity Level [42] was calculated for each of the 10 preferred species for their popularity according to the key informants who cited them in the treatment of particular ailments. Fidelity Level (FL) = Ip/I_u_ x 100 %, where I_p_ is the number of informants who suggested the use of a species for the same major ailment, I_u_ is the total number of informants who mentioned the species for any use.

## Results

### Medicinal plant uses

The communities around Mabira CFR use diverse flora in treatment of various ailments and local people possess rich traditional knowledge on medicinal plants (Table 1). Both males and females used medicinal plants but males were dominant representing 70 % of the respondents. The age of the respondents ranged between 25–80 years. Generally 46 % of the respondents were below 50 years.

**Table 1:** Medicinal plants, their habit, parts used, ailments treated, habitat, method of preparation and administration

Family, scientific name voucher No.	Local name	Habit	Part used	Habitat	Ailment	Method of preparation and administration
ACANTHACEAE						
*Acanthus pubescens* Engl. PT01	Matovu	S	R	F	Prolonged embryo in uterus	Decoction drunk
			L		Measles	Crush in water and bathe
*Asystasia gangetica* (L.) T. Anderson PT242	Ttemba	H	F	FL	Reduce fever in children	Crush and bathe
*Justicia betonica* L.	Kwiniini omuganda	H	L	FL	Weakness in pregnancy	Crush in water and bathe
PT22					Malaria	
			R		Hernia	Decoction dunk
					Worm infection	Pound add water and drink
*Justicia heterocarpa* T. Anderson PT56	Kalaaza	H	L	F	Bad odour in women	pound add to water and wash private parts
					Energy booster in pregnancy	Crush leaves in cold water and bathe early morning
*Thunbergia alata* Sims	Kasaamusamu	C	L	FL	False teeth	Pound and smear at the point of emergence of false teeth
PT28						
ALLIACEAE						
*Allium sativum* L.	Katunguluccumu	H	B	C	Reduce heart beat	Chew and swallow
PT107					Blood cleanser	
					Bad breath	
					Stomachache	
					Constipation	
					Snake bites	Smear at the point of the bite.
					Swollen rib cage	Cut and smear
ALOEACEA						
*Aloe vera* *(*L.*)* Burm.f. PT108	Kigagi	H	L	C/F	Stomachache	1-3 leaves boiled, decoction drunk
					Malaria	
AMARANTHACEAE						
*Achyranthes aspera* L.	Mutassuka kkubo	H	L	F/G	Swollen body Delayed walking in children	Crush and tie on affected part
PT50					Itching body	
						Pound add water and bathe
*Aerva lanata* (L.) Juss. ex Schult PT73	Lweza	H	W	FL	Body odour	Crush in water and bathe
*Amaranthus dubius* Mart. ex. Thell. PT 109	Doodo	H	L	FL	Constipation	Steam and eat
					Anemia	
*Amaranthus spinosus* L. PT243	Doodo owamagwa	H	L	FL	Fungal infections of the scalp	Pound with leaves of *Cleome gynandra* and smear on the scalp
*Celosia trigyna* L. PT110	Kakubaggiri	H	L	FL	Persistent headaches	Rub on the head or Pound, dry, make cuts on the sides of the head and smear
*Psilotrichum elliotii* Bak.	Kanamukasa	H	L	F	Weakness in Pregnancy	Crush in cold water and bathe
PT14						
					Wounds	Boil leaves and place on wound.
					Stomach upsets	Pound add water and drink
ANACARDIACEAE						
*Mangifera indica* L.	Muyembe	T	B	C/F	Cough in children	Decoction drunk
PT111					Infertility in women	
			L		Convulsions	Steaming
*Pseudospondias microcarpa* (A. Rich.) Engl. PT112	Muziru	T	B	F/C	Yellow fever	Pound, decoction drunk
			R		Diarrhoea	
*Rhus vulgaris* Meikle	Kakwansokwanso	S	L	F	Skin rash	Crush, add water and bathe
PT113			R		Erectile dysfunction	Decoction drunk
APIACEAE						
*Centella asiatica* (L.) Urb. PT52	Mbutani	H	L	F	Ulcers	Decoction drunk
APOCYNACEAE						
*Alstonia boonei* De Wild.	Mubajangalabi	T	B	F	Malaria	Decoction drunk
PT120						
*Carissa edulis* (Forssk.) Vahl PT115	Nyonza	S	R	F	Toothache	Pound, boil and press on tooth
ARISTOLOCHIACEAE						
*Aristolochia elegans* Mast PT114	Nakasero	V	S	W	Malaria	Steeped in water and drunk
ASCLEPIADACEAE						
*Mondia whitei* (Hook.f.) Skeels PT121	Mulondo	S	R	F/G	Erectile dysfunction	Chewing
					Low appetite in sickness	
ASTERACEAE						
*Ageratum conyzoides* L.	Nnamirembe	H	L	FL	Weakness in pregnancy	Crush and mix with water and bathe
PT66					Worm infection	Crush and mix with water and drink
*Bidens pilosa* L. PT116	Ssere	H	L	FL	Wounds	Crush, Tie on wound and cut to stop bleeding
					Fresh cuts	
*Conyza adolfi-fridericii* (Musch.) Wild PT117	Ekarwa	H	L	FL	Eye infections	Decoction drunk
*Conyza sumatrensis* (Retz.)	Kafumbe omusaja	H	L	FL	Ringworms	Crush, add paraffin
E. Walker					Wounds	
PT07					Convulsions	Boil and steam the face
*Crassocephalum picridifolium* (DC.) S. Moore PT26	Kitonto	H	L	FL	Weakness in pregnancy	Crushed in cold water and bathed
*Dicrocephala integrifolia* (L.F.) Kuntze	Buzza	H	L	F	Wounds	Crush and Press on the wound or boil.
					Boils	
PT64					Pain in fallopian tubes	Pounded, dried, mixed with water & Drunk
*Erlangea tomentosa* (Oliv. & Hiern) S. Moore PT118	Kisula	H	L	G	Toothache	Crush & press on the tooth
*Helichrysum* sp*. Mill*	Nakabululu	H	L	G	Centipede bites	Crush, mix with salt & rub on the bitten area
PT119						
*Melanthera scandens*(Schumach. & Thonn.) Roberty	Makaayi	H	L	F	Stomachache	Decoction drunk
					Malaria	
					Yellow fever	
PT65					Body odour	Crushed in water & bathed
*Microglossa angolensis* Oliv. & Hiern	Kafuga nkande	S	L	F	Reduce menstrual flow	Pound add water and drink
					Weakness in pregnancy	
					Headache	
PT37					Convulsions	Crush and bathe the child
*Sigesbeckia orientalis* L.	Seziwundu	H	L	F	Fresh cuts	Crush & tie on the cut
PT122					Stomachaches	Decoction drunk
*Sonchus oleraceus* L. PT123	Kakovu	H	L	FL	Scars	Crush and rub on the scar
*Tagetes minuta* L.	Kawunyira	H	L	F,FL,G	Headache	Pound, mix with paraffin and rub on head
					Flu	
					Imperforate vagina	
					Convulsions	Pound, mix with water and wash the birth canal
PT76					Blotting	Crush and inhale
*Vernonia amygdal*ina Delile	Mululuza	S	L	F	Malaria	Crash, add water and drink
PT124			R		Convulsions	
					Stomachache	
*Vernonia auriculifera* Hiern	Kikokooma	S	R	F	Prolonged embryo in uterus	Roots chewed
PT90			L		Weakness in pregnancy	Crush in water and bathe
*Vernonia grantii* Oliv.	Etwatwa	S	L	G	Flu	Steam bathe
PT125					Skin rash	
					Infections	Squeeze into the ear
*Vernonia lasiopus* O. Hoffm.	Kaluluza	S	L	F	Malaria	Crush and mix with cold water and drink
					Stomachache	
PT101					Cough	
			R		Headache Migraine	Pound and drop in the nose.
					Delayed delivery	Burn and chew
BALANITACEAE						
*Balanites aegyptiaca* (L) Delile	Liggwa limu	T	L	G	Yellow fever	Decoction drunk
PT126			R		Diarrhoea	Mixed with *Citrus limon* leaves, boiled and drunk
					Wounds	
					Skin rash, Flu	Boil & wash
					Paronychia	Crush and tie on finger
			B		Impotency	Decoction drunk
*Balanites wilsoniana* Dawe & Spraque PT130	Naliggwalimu	T	L	F	Cracks of soles of feet	Crush and smear on feet
BASELLACEAE						
*Basella alba* L.	Nderema	H	L	F	Stomachache Constipation	Dry, pound and add to sauce
PT128					Prolonged embryo in uterus	
BIGNONIACEAE						
*Kigelia africana* (Lam.) Benth.	Mussa	T	B	F	Stress	Decoction drunk & bathed
					High blood pressure	
					Impotency	
PT127			L		Loss of appetite	Decoction drunk
*Markhamia lutea* (Benth.) K. Schum	Musambya	T	FL	F	Ear & eye infections in children	Pound and drop in the ear or eye
PT129			L		Malaria	Decoction drunk
					Hoarse voice	Chew
*Spathodea campanulata*	Kifabakazi	T	L	F	Pregnancy care	Crush add water & bathe
P. Beauv.			B		Increase vaginal fluids	Pound, decoction drunk
PT131			R		Infertility	
					Skin infection	Boil and bathe
					Hernia	Decoction drunk
BRASSICACEAE						
*Cardamine trichocarpa* Hochst. Ex. Rich.	Mageregankoko	H	L	FL	Athletes foot	Burn and squeeze on the feet
					Ringworms	Boil and bathe
PT132					Immobility in children	
BURSERACEAE						
*Canarium schweinfurthii* Engl. PT133	Muwafu	T	B	F	High blood pressure	Decoction drunk
					Diabetes	
					Cough	
CANELLACEAE						
*Warburgia ugandensis* Sprague PT136	Barwegyira	T	B	F	Flu	Decoction drunk
					Cough	
CANNABACEAE						
*Cannabis sativa* L.	Njaga	H	L	C	Measles	Decoction drunk
PT135					Body weakness	
CAPPARACEAE						
*Cleome gynandra* L.	Jjobyo	H	R	FL	Ease delivery Fungal skin infections on head	Chew the roots
PT134						Mix in sheep dung and smear on the affected parts
*Cleome monophylla* L.	Kayobyo akasaja	H	FL	FL,W	Retained placenta	
PT137						
CARICACEAE						
*Carica papaya* L.	Mapapali	H	L	C/F	Cough	Dry, pound, mix in water and drink
PT138					Low immunity	
					Cracks on soles of feet	Scrub on the soles of feet
					Skin infection	Pound mix with water and bathe
					Loss of memory	Burn and smell
			L		Measles	Pound add water and bathe
			R		Erectile dysfunction	Pound add water and drink
*Elaeodendron buchananii* Loes.	Mbaluka	T	B	F	Blocked fallopian tube	Decoction drunk
					Prostate cancer	
PT121					Erectile dysfunction	
CHENOPODIACEAE						
*Chenopodium opulifolium* Koch & Ziz	Mwetango	H	L	FL	Oral wounds	Chew mixed with salt
PT83					Skin rash	Pound, add little salt put on tooth
					Toothache	
					Sore throat	Squeeze in mouth and swallow
*Chenopodium procerum* Hochst. ex Moq. PT37	Mugoosola	H	L	FL	Weakness during pregnancy	Herbal bath
CLUSIACEAE						
*Psorospermum febrifugum* Spach	Kanzironziro	S	L	W	Skin rash	Pound, dry, mix in Vaseline and smear
PT139			R		Dry cough	Pound, decoction drunk
					Wounds	Pound, mix with water and bathe
*Garcinia buchananii* Baker PT140	Musali	T	R	F	Hurting bones	Pound add to tea
					Diabetes	
*Harungana madagascariensis* Lam. ex Poir.	Mulirira	S	B	F	Yellow fever	Pound add to water and bathe
PT210						
COMBRETACEAE						
C*ombretum molle* R. Br. G. Don	Ndagi	T	B	G	Cough	Decoction drunk
PT03						
COMMELINACEAE						
*Commelina benghalensis* L.	Nnanda	H	L	F	Vaginal dryness	Pound , mix with water and wash private parts
PT145					Weakness in sickness	Pound, add water and bathe
					Abortion	
CONVOLVULACEAE						
*Ipomea batatas* (L.) Lam.	Lumonde	C	T	C	Memory loss	Chew
PT141					Paronychia	Burn and pound and tie on the finger
*Hewittia sublobata* L. Kuntze	Musota taluma	C	V	F/G	Pregnancy care(widens pelvic girdle)	Tie in the waist
PT239					Headache	Smear on head and bitten part
			T		Snake bites	
			L		Persistent headache	Crush and smear on the head
CRASSULACEAE						
*Kalanchoe crenata* (Andrews) Haw.	Kayondo akatono	H	L	FL	Healing umbilical cord wounds in babies	Place on fire & squeeze onto the cord
PT143						Crush, add water and bathe
					Skin rash in babies	Crush add water & drink
					Cough	Herbal bath
						Pound mix with water and drink
*Kalanchoe glaucescens* Britten	Kiyondo	H	L	FL/G	Cough	Crush and drink
PT142					Break cords from new borns	Put the leaves on fire and squeeze on the cord
CUCURBITACEAE						
*Kedrostis foetidissima* (Jacq.) Cogn.	Ziizi (kabaka wenva)	V	W	F	Measles in children	Mix with silver fish and boil and drink
PT205			L		Loss of appetite	Boil and add to sauce
*Mormodica feotida* Schumach	Lujjula (bombo)	V	L	F	Body odour	Pound , mix with water and bathe
PT144						
DRACAENACEAE						
*Dracaena fragrans* (L.) Ker. Gawl. PT149	Mulamura	S	B	F	Tooth ache	Chew and spit
			R		Rheumatism	Pound and drink
*Dracaena steudneri* Engl.	Kajolyenjovu	T	L	F	Cough	Burn the leaves and collect the ash add salt and lick
PT146			B		Scars	Pound the bark, mix with ghee, smear on the scar
					Snake bites	Pound and press on the bitten part
					Syphilis	Decoction drunk
		R			Skin infections	Pound mix with water and bathe
					Kidney stones	Pound ,decoction drunk
		FL			To stop smoking and alcoholism	Pound, dry add little water and drop in a cigarette or alcohol
EBENACEAE						
*Diospyros abyssinica* (Hiern) F. White PT147	Mpojja	T	L	F	Stomach upsets	Decoction drunk
EUPHOBIACEAE						
*Acalypha bipartita* Müll. Arg. PT148	Jerengesa	S	L	F	Constipation	Crush, add water and drink
*Alchornea cordifolia* (Schumach. & Thonn.) Müll. Arg. PT06	Luzibaziba	S	L	F	Shaking body	Crush and bathe
*Croton macrostachyus* Hochst. ex. Delile	Musogasoga	T	L & R	F	One stuck by lightening	Pound add to water and bathe
PT240			V		Weakness in pregnancy	Tie in the waist
						Tie on the head
			T		Headache	Pound and smear on the bite
					snake bites	
*Euphorbia hirta* L.	Kasandasanda	H	S	FL	Swollen eyes	Drop the sap in the affected eye.
PT150			L		Joint pains	Pound, dry , mix with Vaseline and smear on the joints
*Euphorbia trigona* Haw.	Kakukulo	S	L	F	Yellow fever	Pound mix with ghee and maize flour and smear body
PT151					Skin allergy in children	Pound and to water and bathe
			S		Backache	Cut and release the sap on the cut.
*Eurphobia tirucalli* L.	Lukoni/nkoni	T	L & S	C	Warts	Drop the sap on the wart
PT152						
*Flueggea virosa* (Roxb.ex Willd.) Royle. PT17	Lukandwa	S	R	F	Infertility in women	Pound add to water and bathe
*Hymenocardia acida* Tul.	Nabaluka	T/S	L	W	Sinuses	Decoction drunk
PT153						
*Jatropha curcas* L.	Kirowa	S	L	C	Tooth decay	Crush and drop sap on tooth
PT160					Headache	Crush, add water & wash the head
					Weakness in pregnancy	Crush & Bathe in cold water
*Margaritaria discoidea* (Baill). G.L. Webster	Kamenyambazi	T	B	F	Oversleeping	Decoction drunk
PT161						
*Ricinus communis* L.	Nsogasoga	S	L	C,F	Weakness in pregnancy	Poundadd to water and bathe
PT154			R		Ear infection	Pound add drop in the ear
*Tetrochidium didymostemon* (Baill.) Pax & K. Hoffm PT155	Mukejje	T	L	F	Measles	Crush add to water and drink
*Tragia benthamii* Baker	Kamyu	H	R	G	High blood pressure	Pound , dry and add to tea
PT40					Erectile dysfunction	Chew
			L		Madness	Pound ,cut in the head and smear
FABACEAE						
*Abrus precatorius* L	Lusiiti	C	L	W/FL	Low immunity	Decoction drunk
PT162			R		Worm infection	Chew and swallow
*Acacia constricta* Benth.	Muwelamanyo	T	R	FL	Diabetes	Decoction drunk
PT163					Sinuses	Steam bathe
					Convulsions in children	
*Acacia hockii* De Wild.	Kasaana	T	R	W,G	Swollen joints and feet	Pound, boil with cows hooves and drink soup
PT18						
*Acacia macrothyrsa* Harms PT156	Muwologoma	T		W	Hydrocele	
*Acacia siberiana* (DC.) Kyal. & Boatwr.	Muwawa	T	B	W	Sinuses	Decoction drunk
PT157			R		Convulsions in children	Herbal bathe
*Albizia coriaria* Welw.	Mugavu	T	B	F	Skin rash	Boil and bathe
PT158					Cough in children.	Decoction drunk
					Swollen rectum	Boil and sit in the water
*Albizia grandibracreata*	Nongo	T	L	F	Yellow fever, Anaemia	Pound, dry and mix with water and drink
PT60			B		Fungal infections of the scalp	Pound inner bark, mix in water and wash the head
*Alysicarpus vaginalis* (L.) DC. PT31	Nakalimikamu	T	L	FL	Irregular menstrual periods	Decoction drunk
*Mimosa pudica* L.	Wewumbe	H	L	G/F	Treat children that have failed to walk.	Crush and smear on joints
PT164						
*Crotalaria agathiflora* Scheinf. ex Engl. PT165	Kijebejebe	S	L	FL	Low breast milk production	Mix leaves with fresh simsim, boiled & drunk
*Crotalaria natalitia* Meisn PT166	Tulo	S	L	FL	Nightmares	Burn and inhale smoke
*Crotalaria spinosa* Hochst.	Kasambandege	H	L	FL	Weakness in pregnancy	Crush and mix in water and drink Crush in water and bathe
PT170					Skin itching	
					Convulsions	
					Prolonged embryo in uterus	Pound a few leaves mix with water & drink
					Constipation	
*Dichrostachys cinerea* Wight et. Arn. PT159	Muwanika	S	R	G	Hutch bark	Decoction in early stages of the condition drunk
*Erythrina abyssinica* Lam.	Jjiirikiti	T	B	F/G	Yellow fever	Decoction drunk
PT167					Convulsions	Pound, add salt, put in a clean cloth and squeeze in the mouth
					Anaemia	
					Infertility in women	
					Hicupp	
					Vomiting	
*Entada abyssinica* Steud. ex A. Rich.	Mwoloola	T	B	W	Body weakness	Boil in water and bathe when cold
PT168			L		Oral wounds	Chew with salt
					Skin infections, fresh cuts and wounds	Crush, rub and tie on affected part or wound
					Change sex of children	Concoction boiled and drunk
*Indigofera arrecta* Hochst. A. Rich PT81	Kabamba maliba	H	L	F	Snake bites	Pound, add water
					Wounds	Crush & tie on wound
*Indigofera congesta* Welw.ex. Baker	Namasumi	H	L	G	Malaria	Decoction drunk
PT169						
*Indigofera drepanocarpa* Taub.	Sebazinga nkata	H	S	G	Colic pains	Sap ingested
PT14			W		Convulsions	Tie in the waist
*Indigofera emarginella* Steud. ex A. Rich. PT170	Katungansozi	H	R	G	Elephantiasis	Pound, mix with vaseline and smear
*Indigofera spicata* Forssk.	Mukaliza	H	L	G	Vaginal discharge	Crush in water and wash private parts
PT02						
*Piptadeniastrum africanum* (Hook F.) Brenan PT59	Mpewere	T	L	F	Cough	Steam bathe
*Rhychosia hirta* (Andr.) Meikle & Verdc. PT171	Katinvuma	C	L	F	Herpes zoster	Crush and smear on affected parts
*Senna absus* (L.) Roxb.	Mucuula	S	L	F	Prolonged embryo in uterus, Malaria	Pound add water and drink
PT172						
*Senna didymobotrya*(Fresen.) H.S. Irwin & Barneby	Mukyula	S	L	F	Change sex of children	Pound, decoction drunk
PT180					Stomachache	
*Sesbania sesban* (L.) Merr.	Muzimbandeya	S	R	F	High blood pressure	
PT185					Diabetes	
*Tamarindus indica* L.	Mukooge	T	R	W/F	Convulsions	Steam the face
PT186			FR			
			L		Stomachache	Decoction drunk
*Vigna unguiculata* L.	Kiyindiru	H	L	F/G	Sore throat	Add salt and chew
PT173						
FLACOURTIACEAE						
*Dovyalis macrocalyx* (Oliv. J. Warb) PT61	Mutunku	S	L	F	Wounds	Crush & tie on wound
LAMIACEAE						
*Coleus latifolius* Hochst. Ex. Benth. PT38	Mubiru	H	L	G	Vaginal dryness	Steam and insert in birth canal
*Clerodendrum myricoides* (Hochst.) R. Br.Vatke PT55	Kikonge	T	R	G	Stomachache	Pound add water and drink
*Hoslundia opposita* Vahl	Kamunye	H	L	F,G	Painful uterus	Decoction drunk
PT89					Stomach cleanser	
					Malaria	
					Fresh cuts	Crush and squeeze on the cut and tie around the cut.
					Skin rash	Pound, dry add to Vaseline and smear
*Leonotis nepetifolia* (L.) R	Kifumufumu	H	L	F	Abdominal pain	Decoction drunk
Br. PT174					Kidney stones	
					Body pains(muscles)	Crush + paraffin and smear on painful parts
*Mentha* Sp*.*	Nabugira	H	L	F	Body odour	Crush in water and bathe
PT175						
*Ocimum basilicum* L.	Kakubansiri	H	L	F,W	Stomachache	Pound, add water and drink
PT82					Pain during pregnancy	Crush and smear
					Prevent miscarriage	
					Insect bites	
*Ocimum gratissum* L.	Mujaja	H	L	FL	Stomachache	Decoction drunk/boiled in tea and drunk
PT176					Bad breath	Squeeze leaves in cold water and bathe
					Kwashiorkor	
*Plectranthus barbartus* Andr. PT57	Kibwankulata	H	L	F	Wounds	Crush and tie on wound
*Tetradenia riparia* (Hochst.) Codd	Kyewamala	T	L	C	Cough	Crush, mix with water and drink
PT178					Stomachache	Squeeze the leaves and drop in ear or eye
					Eye & ear infections	Pound mix in water and bathe
					Weakness in pregnancy	
LAURACEAE						
*Persea americana* Mill.	Avacado pear	T	B	C/F	Cough	Decoction drunk
PT179						
LOGANIACEAE						
*Strychnos innocua* Del.	Muyondo	S	L	W	Athletes foot	Heat on fire & press on affected area
PT181					Tooth decay/pain	Boil and mix with salt and press on tooth
MALVACEAE						
A*butilon mauritianum* (Jacq.) Medik. PT42	Kifuula	H	L	W	Change sex of children	Squeeze in water and drink before getting pregnant
*Hibiscus acetosella Welw*. Ex Fic PT23	Musaayi	S	L	FL	Anaemia	Decoction drunk
*Sida alba* L.	Keyeyo	H	L	W	Fractures	Pound, smear on swollen body with or without Vaseline
PT182					Swollen body	
*Sida cuneifolia* Roxb.	Kakumirizi	H	L	FL	Fractures	Crush and Press on the affected area
PT53					Pain the fallopian tubes	Decoction drunk
					Fever	herbal bathe
*Sida rhombifolia* L. PT09	Luvunvu	S	R	F	Lack of breast milk	Boil with silver fish and drink
MELASTOMATACEAE						
*Tristemma maritiana* A. Juss. PT97	Musesemya	H	L	F	Enable one to eat meat or fish	Pound, dry and add to sauce
MINESPARMACEAE						
*Cissampelos mucronata* A. Rich.	Kavamagombe	S	L	G	Weakness in pregnancy	Pound, add to water & bathe
PT63					Backache	
					Snake bites,	Pound leaves and tie on affected part
					Swollen legs	
			R		Aching bones	
					Stomachache	Pound add water & drink
MORACEAE						
*Antiaris toxicaria* Lesch.	Kilundu	T	L	F	Headache	Crush in water and bathe
PT183					Weakness in pregnancy	
*Ficus cyathistipula* Warb.	Mubembe	S	L	F	High blood pressure	Decoction drunk
PT99						
*Ficus dawei Hutch*.	Muwo	T	B	F	Breast cancer	Decoction drunk
PT184					Wounds	Dried powder applied to the wound
*Ficus mucuso* Welw. ex Ficalho PT186	Kabalira	T	L	F	Swollen eyes	Pound, burn and press on the eye
*Ficus natalensis* Hochst.	Mutuba	T	B	F	Gonorrhea	Decoction drunk
PT187						
*Milicia excelsa* (Welw.) C.C. Berg	Muvule	T	B	F	Skin rash	Boil and bathe
PT188			S		Burns	Pour sap on burn area
					Fresh cuts	Smear the sap on the cut
*Myrianthus arboreus* P. Beav. PT195	Mugango	S	R	F	Control pregnancy	Tie on the waist
MORINGACEAE						
*Moringa oleifera* Lam.	Muringa	T	FL	C	Aching joints	Pound , dry sieve, mix with Vaseline and smear on joints
PT189						
MUSACEAE						
*Musa paradisiaca* L. *var paradisiaca* PT190	Kitooke ekiganda	H	FL	C	Prolonged embryo in uterus	Pound the sheath & chew
			R		Swollen legs	Chew the roots
			S		Sternum pain	Pound and smear on swollen or painful part
*Musa paradisiaca* L. var *sapientum*	Gonja	H	F	C	Neck pain	Tie the fiber in the neck and waist
PT191					Control pregnancy	
			FR		Umbilical cord wounds	Scrape and put on cord
			R		Induce labour	Place in fire and chew
MYRICACEAE						
*Morella kandtiana* (Engl.) Verdic & Polhill	Mukikimbo	S	R	F	Stomachache	Crush in cold water and drink
PT192					Snake bites	Chew and smear at the site of the bite
					Hernia of the heart	Chew and swallow
MYRTACEAE						
*Callistemon citrinus* (Curtis) Skeels	Mwambala zitonya	T	L	C	Pain in the Fallopian tubes	Decoction drunk
PT88					Cough	
*Eucalyptus* sp	Kalituunsi	T	B	C	Cough	Decoction drunk
PT193			L		Boils	Mix with 10 seeds of Jackfruit and leaves of *Erythrina abyssinica* and mix in 4 cups of water and boil to 3 cups, drink
*Psidium guajava* L. PT200	Mupeera	T	L	C	Cough	Decoction drunk
*Syzgium cumini* L. PT201	Jambula	T	L	C	Cough	Decoction drunk
*Syzygium cordatum* Hochst. PT194	Kanzironziro	T	L	C/F	Skin rash	Crush and mix in Vaseline and smear
			R		Dry cough	Pound, decoction drunk
					Wounds	Pound, mix with water and wash wound
MYRSINANCEAE						
*Maesa lanceolata* G. Don	Kiwondowondo	T	R	F	Ulcers, Diarrhoea	Decoction drunk
PT04			L		Convulsions	Herbal bathe
OXALIDACEAE						
*Oxalis corniculata* L.	Kajjampuni	H	L	FL	Wounds	Squeeze and drop juice on wounds.
PT195					Athletes foot	Place on fire and place on toes
					Skin cancer	Pound, dry and put on the wound
					High blood pressure	Chew the leaves
					Diabetes, Hormonal imbalance	
PASSIFLORACEA						
*Passsiflora edulis* Sims	Katunda	C	FR	C/F	Weakness in sickness	Squeeze juice, add water and drink
PT196						
PHYLLANTHACEAE						
*Phyllanthus guineensis* Pax	Mutulika		L	F	Measles	Crushed in water and bathed
PT87						
PHYTOLACACEAE						
*Phytolaca dodecandra* L’Hér.	Luwoko	S	L	F	Skin rash	Pound, mix in water and bathe
PT197			R		Swollen joints	Crush in water and bathe
			FR/S		Cracks on the soles of the feet	Crush and smear on the feet soles
PLANTAGONIACEAE						
*Plantago palmata* Hook.f.	Bukumbu	H	R	F	Skin rash in children	Crush in water and bathe
PT85						
POACEAE						
*Arundinaria alpina* K. Schum. PT198	Mabanda	G	R	F	Fainting/Epilepsy	Pound and bathe
					Skin rash	
*Cymbopogon citratus* (DC) Stapf PT199	Kisubi	G	R	G	Pain in fallopian tubes	Decoction drunk
*Cymbopogon nardus* (L.) Rendle PT91	Kitete	G	R	G	Eye infection	Pound, dry add to eyes
					Pain in fallopian tubes	Pound add water & drink
*Cynodon dactylon* (L.) Pers. PT44	Kalandalugo	G	S	G	Prolonged embryo in uterus	Decoction drunk
					Painful breasts	
*Digitaria abyssinica (A. Rich.) Stapf*	Lumbugu	G	W	G	Convulsions	Cut boil and steam
PT202			L		Flu	
					Diarrhoea	Decoction drunk
*Imperata cylindrica* (L.) P. Beauv. PT203	Lusenke	G	R & L	G	Snake bites	Chew roots and tie leaves at the site of the bite
*Pennisetum purpureum* Schumach. PT204	Kisagazi	G	L	F	Penile erection in baby boys	Crush in water and wash the penis
POLYGONACEAE						
*Rumex abyssinicus* Jacq.	Muleretu	H	R	G	Erectile dysfunction	Chewing
PT135					Low appetite after sickness	
*Oxygonum sinuatum* (Meissn.) Dammer	Kafumita bagenge	H	L	FL	Wounds	Pound and tie around the affected finger
PT67					Paronychia & boils	Mix with ghee and rub on affected joints
					Painful joints	
*Polygonum setosulum* A. Rich PT206	Kifumita bagenda	H	L	FL	wounds	Pound and tie around the affected finger
					Paronychia	
PORTULACACEAE						
*Portulaca oleracea* L.	Ssezira	H	L	FL	Irregular menstrual periods, Stomachache	Decoction drunk
PT207						
PRIMULACEAE						
*Primula sieboldii* E. Morren PT208	Muyuki	H	B	F	Tonsillitis	Decoction drunk
					Ulcers	
RHAMNACEAE						
*Maesopsis eminii* Engl.	Musizi	T	R	F	Syphilis	Decoction drunk
PT209						
ROSACEAE						
*Prunus africana* (Hook.f.) Kalkman PT220	Ngwabuzito	T	L	F	Fainting	Decoction drunk
					Prostate cancer	
*Rubus pinnatus* willd	Nkenene	S	FR	F	Energy booster	Eat fresh
PT238						
*Rubus rigidus* Sm	Kawule	S	R	F	Stomach upsets	Decoction drunk
PT79			L		Skin rash	Pound, dry mix with Vaseline and smear
					Snake bites	Crush and tie on affected area.
RUBIACEAE						
*Coffea eugenioides* S. Moore	Mwanyi	S	FR	F	Erectile dysfunction	Roast and chew
PT221					Oversleeping	
			R		Erectile dysfunction	Chew
			S		Heart burn	
*Mitragyna stipulosa* Kuntze PT230	Nzigu	T	L	F	prolapsed rectum	Pound place sap on rectum and tie some leaves on.
*Rubia cordifolia* L.	Kasarabakesi	C	L	F	Cough	Pound with onions, add salt & Lick
PT25					Tuberculosis	Dry, burn & lick the ash
*Vangueria apiculata* K. Schum. PT222	Matugunda	S	R	F	High blood pressure	Decoction drunk
					Hiccups	
RUTACEAE						
*Citropsis articulata* Swingle & Kellerm. PT223	Katimbolo	S	L	F	Impotence	Decoction drunk
			B			
*Citrus limon* (L.) Osbeck.	Nimawa	T	FR	C/F	High blood pressure	Juice drunk
PT229					Cough	
					Blotting	
					Skin rash/pimples	Add to water and wash the affected parts
						Chop, decoction drunk
					Sore throat	Chew
					Nausea during sickness	
*Citrus sinensis* (L.) Osbeck	Muchungwa	S	L	C/F	Bad breath	Chew
PT228						
*Teclea nobilis* Del. PT227	Nzo	T	L	F	Body cleanser	Boil with *afromomum* and drink
*Zanthoxylum chalybeum* Engl. PT224	Ntale ya ddungu	T	R	F/W	Cervical cancer	Pound, add water & drink
			B		Stomachaches	
					Cough	Decoction drunk
SAPINDACEAE						
*Blighia unijugata* Baker	Mukuzanyana	T	B	F	Cervical cancer,	Decoction drunk
PT29					Fibroids	
SOLANACEAE						
*Capsicum frutescens* L.	Kamulali	H	FR	C/F	Hernia, Pancreas	Swallow the fruits
PT225					Prostate cancer	Eat in food
			R		Erectile dysfunction	Pound, add water and drink
*Datura stramonium* L.	Kituratura	H	R	FL	Failure to walk in children	Pound roots, put under fire and press the feet of the child
PT226						
*Lycoperscon esculentum* (L.) H. Karst	Nyanya	H	L	FL	Skin infections	Herbal bathe
PT231			FR		Anaemia	Eat raw
					Kidney stones	
*Nicotiana tobaccum* L	Taaba	H	L	C/FL	Snake bites	Chew and vomit the venom
TP232					Paronychia	Tie on the affected finger.
*Physalis peruviana* L.	Ntutunu enene	H	L	F	Fainting	Smear whole body
PT236			FR		Ear & Eye infection	Chew and swallow
*Solanum anguivi* Hook	Katunkuma	H	FR	C/F	Measles	Pound ripe fruits, smear whole body
PT237					High blood pressure	Boil, pound and dry, add to food
					Weakness during sickness	Steam and eat as a vegetable
					Blood cleanser	
*Solanum dasyphyllum*	Ntengontengo	S	FR	FL	“Elongation of the labia minora	Roast in fire, peel of the outer parts, use endocarp.
Schumach. & Thonn.						
PT41			R		Warts	Boil and place on the wart.
					Immobility in babies	Place in fire and place on the child’s feet
					Swollen stomach	Decoction drunk
*Solanum incanum* L.	Katengo ntengo	H	R	FL	Erectile dysfunction	Chew
PT49					Swollen testicles	Pound, add water and drink
					Flu	
			FR		Headache	Smear on the head
*Solanum micranthum* Schltdl.	Katuntunu	H	L	F	Bed wetting	Pound leaves, mix in water and drink
PT27						
					Irregular menstrual periods	Crush , add water and bathe
					Itching vagina, Skin rash	Squeeze into the ear
					Ear infections	
*Solanum nigrum* L.	Nsuga nzirigavu	H	L	F	Low immunity	Prepare as vegetable
PT68			S		Pain in fallopian tubes	Crush, boil & drink
					Malaria	
					Stomachache	Drink or eat as vegetable
VERBENACEAE						
*Lantana trifolia* L.	Kayukiyuki	S	L	F	Prolapsed rectum	Pound and place on affected part
PT05			R		Ring worms	
					Yellow fever	Pound decoction drunk
					Painful muscles	
					Bloating stomach	Pound add water and drink
*Priva flabelliformis* (Mold.) R. Fernand	Nkami	H	S	G	Wounds	Release the sap onto the wound
PT233			L		Diarrhoea	Pound leaves add water and drink
VITACEAE						
*Cyphostemma adenocaule* (A. Rich) Willd & Drummond PT58	Kabombo	H	W	F	Body odour	Crush in water and bathe
					Constipation	Crush in water and drink
			L		Measles	Decoction drunk
					Syphilis	Crush mix with water and bathe
ZINGIBERACEAE						
*Afromomum anguistifolium* (Sonnerat) K. Schum.	Matungulu	H	R	F	Hiccup	Dry, pound, decoction drunk
PT234					Obesity	Pound
			FR		Low immunity	Boil the fruit and drink
*Zingber officinale* Roscoe	Ntangawuzi	H	T	F/C	Cough	Chew and swallow or boil in tea,
PT235					Backache	
					Erectile dysfunction	

Key: Parts used: *R* roots, *L* Leaves, *Fl* Flowers, *W* whole plant, *B* Bark, *Fr* Fruit, *T* Tuber, *S* Sap, *V* Vine ; Habit: *S* Shrub, *T* Tree, *H* herb, *C* Climber, *G* grass; Habitat: *F* forest, *FL* farmland, *C* cultivated, *W* woodland, *G* grassland

A total of 190 plant species distributed in 61families and 152 genera were identified as used. Fabaceae contributed 27 species, followed by Asteraceae (17), Euphorbiaceae (13), Solanaceae (10) and Lamiaceae (9). Genera *Solanum* and *Indigofera* contributed five species each while *Ficus, Vernonia,* and *Acacia* contributed four species each.

### Preferred medicinal plant species

*Vernonia amygdalina* was highly ranked and regarded most important in treatment of malaria in the study area. Table 2 shows ranking of the ten most important plant species according to key informants in decreasing order together with values assigned by each informant. The key ailments treated by the preferred medicinal plants were mentioned by the key informants during the interviews.

**Table 2:** Rank values assigned by each informant for each of the 10 preferred medicinal plants

Medicianl plant species	Plant parts used	Key ailments treated	Key informants (*n* = 12)												value/120	Rank
			A	B	C	D	E	F	G	H	I	J	K	L		
*Vernonia amygdalina*	Leaves, Roots	Malaria, Convulsions, stomachache	10	10	10	10	10	10	10	10	10	10	10	10	120	1^st^
*Mormodica feotida*	Leaves	Body odour	8	9	9	9	9	8	7	8	7	6	7	8	95	2^nd^
*Warbugia ugandensis*	Bark	Cough, flue	5	8	7	7	8	9	9	6	5	7	9	9	89	3^rd^
*Prunus africana*	Leaves, Bark	Fainting, prostate cancer	9	5	8	8	7	7	8	4	9	8	8	7	88	4^th^
*Piptadeniastrum africana*	Leaves, Bark	Cough	7	7	6	6	5	4	5	7	8	9	5	6	75	5^th^
*Erythrina abyssinica*	Bark	Yellow fever, convulsions, anaemia, infertility hiccup, stop vomiting	6	6	5	4	6	6	6	9	6	5	2	5	66	6^th^
*Albizia corriaria*	Bark	Cough, swollen rectum, skin rash	1	4	4	5	4	5	4	5	4	4	6	4	50	7^th^
*Spathodea campanulata*	Leaves, Bark, roots	Pregnancy care, infertility, skin infections, hernia	4	3	3	1	3	2	3	3	2	1	4	3	32	8^th^
*Mondia whitei*	Roots	Stimulate sexual potency, energy booster	2	1	2	3	1	3	2	1	3	3	1	2	24	9^th^
*Alstonia boonei*	Bark	Malaria	3	2	1	2	2	1	1	2	1	2	3	1	21	10^th^

Key scores in the table indicate ranks given to medicinal plants based on their efficacy and availability by informants. Highest number (10) for medicinal plant which informants thought most effective in treating ailments and available and the lowest (1) for the least effective and rare. The criterion for considering key ailments was all aiments that were mentioned by informants during interviews

### Growth forms of Plants and parts used for medicinal purposes

Different plant parts of medicinal plants are used to make herbal preparations (Table 3). A high number of herbal medicine are made using leaves (77 %) and roots 40 %. Other parts of the plants are not commonly used. Regarding the 10 preferred medicinal plant species, the bark was predominantly used in seven species, followed by leaves (5) and least roots (3) (Table 3 ), although more than one part was used in some cases. For instance leaves, bark and root of *Spathodea campanulata* and leaves, roots and fruits of *Tamarindus indica and Phytolaca dodecandra* are used to prepare remedies. Herbs made up the highest proportion of medicinal plants species (41 %), followed by trees (28 %), shrubs (22 %), climbers and grasses (4 %).

**Table 3:** Plant parts used for medicinal purposes

Plant part used	No. of plants species (*n* = 190)	% use
Leaves	147	77.4
Roots	75	39.5
Bark	31	16.3
Fruit	17	8.9
Flowers	6	3.2
Whole plant	8	4.2
Branches	4	2.1
Sap	6	3.2

The figures are inclusive of each other

### Source of medicinal plants

Of the recorded medicinal plants, 56 % are from the forest, 14 % are cultivated 12 % grow in grasslands/woodlands and farmlands (18 %). The low incidence of medicinal plant gardens was attributed to the need to maintain secrecy of traditional knowledge and the argument that cultivated medicinal plants are less potent compared to plants collected from the wild and therefore the latter are preferred. Medicinal plant species from the forest were mostly members of Fabaaceae (40 %) and Euphorbiaceae (54 %) while species from family Asteraceae were dominant in grasslands (25 %) and fallow (44 %). Most of the medicinal plants grown in home gardens are introduced species and have not been domesticated. These include: *Callistemon citrinus, Capsicum frutescens, Moringa oleifera*, plus fruit tree species that are also medicinal such as *Mangifera indica, Persea americana, Carica papaya* and *Psidium guajava.* Fifty percent of medicinal plant users who harvest for commercial purposes collect plants form the forest.

### Methods of preparation and administration

**Fig. 2:**
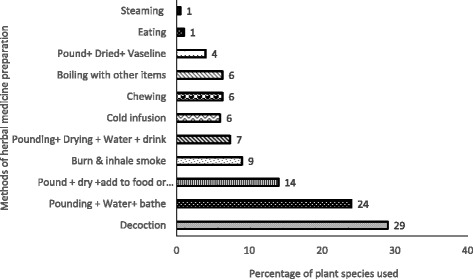
Percentage of species prepared using different methods. The figure depicts the percentage of medicinal plant species used for making herbal remedies using different methods according to information obtained from key informant interviews. The total number of species for calculation of percentages was 190. In some cases herbal remedies from the same medicinal plant species could be prepared using more than one method. The main ingredient used in preparation of herbal remedies was water in the case of decoctions and cold infusions. Method of preparation varied according to the plant species, plant part used and sometimes the condition being treated

The medicinal plants for treatment of different ailments were prepared and administered using various methods. Decoction was commonly used (29 %), followed by crushing and mixing with water (24 %), use of fresh crushed material (14 %) and burning (9 %) (Fig. 2). In the current study, additives used in herbal medicine preparation included silver fish, ash, salt, alcohol, tea and onions. Salt was used in remedies against toothache and oral wounds where it is believed to kill germs. For external application vaseline, paraffin and ghee were used to reduce friction during application of the remedy.

Different routes were used in administration of herbal preparations. Oral route contributed 61 % of the total species, followed by herbal bath (28 %), rubbing leaves on affected parts (14 %) and inhalation of smoke (5 %). The least used route of herbal administration was steam bath (2 %).

### Ailments treated by medicinal plants

The 58 health conditions recorded were grouped into 25 categories of which gynecological conditions, digestive disorders and skin infections featured prominently (Table 4). The number of species used to treat different ailments are summarized in Table 4.

**Table 4:** Ailment categories treated by different medicinal plants

Ailment categories	Specific conditions	No. of species used (*n* = 190)	% of total species
Gynaecological issues	Heavy menstrual flows, weakness during pregnancy, increasing vaginal fluids, uterine cleansing, family planning and induction of labour.	58	30.5
Digestive disorders	stomachaches, blotting, ulcers, constipation, diarrhea, weight loss	54	28.4
Skin infections	Wounds, warts, skin rash, acne, pimples and athletes foot.	47	24.7
Malaria & other infections	Malaria, yellow fever, measles, toothache, ear & eye infections	43	22.6
Respiratory tract infections	Flue, sinuses, sore throat, cough, tuberculosis	34	17.9
Arthritis & inflammation	Swollen body parts, hydrocele elephantiasis. hernia, boils	23	12.1
Neurological & nervous system disorders	Convulsions, epilepsy, fainting	17	8.9
Erectile dysfunction& Impotence	Male sexual vitality	13	6.8
Ailment categories	Specific conditions	No. of species used (*n* = 190)	% of total species
Childcare	Swollen rib cage, failure to walk, umbilical cord treatment, false teeth, colic pains	12	6.3
Poisonous animal bites	Snake and centipede bites	12	6.3
Hypertension	Control of heart beat	11	5.8
Immune & energy boosting	Low appetite, nausea	10	5.3
Painful body parts	Neck, sternum pain,	10	5.3
Body odour	Bad breath,	9	4.7
Headaches & Fatigues	Migraines	6	3.2
Diabetes		6	3.2
Cancer	Prostate, skin, breast and cervical cancer	6	3.2
Blood system disorders	Blood cleansing, anaemia,	5	2.6
Muscular skeletal problems	Back ache, joint pains, Rheumatism, shaking body, fractures	4	2.1
STDs & Venereal diseases	Gornorrhea, syphilis	4	2.1
Abnormalities	Hunchback	3	1.5
Hiccups		3	1.5
Psychiatric disorders	Madness, memory loss, night mares	2	1.1
Bedwetting		1	0.5
Stop smoking		1	0.5

Species treated a wide range of ailments varying from one to six per plant. Species that treated the highest number of ailments were *Balanites aegyptiaca, Carica papaya, Dracaena steudneri* that were used in management of six health conditions each. On the other hand *Allium sativum, Cissampelos macronata, Kalanchoe crenata*, *Lantana trifolia, Solanum anguvi, Tagetes minuta* and *Vernonia lasiopus* were each used in management of five health conditions. Taxonomic analysis revealed that members of family Fabaceae were used to treat the highest percentage (28 %) of ailments. This was followed by Solanaceae (24 %), Asteraceae and Euphorbiaceae (19 %) each, Amaranthaceae, Balanitaceae and Rutaceae 14 % each, Anarcadiaceae, Moraceae, Poaceae, Bignoniaceae 12 % each while families Alliaceae, Caricaceae, Dracaenaceae, Lamiaceae, Minespermaceae, Rosaceae, Rubiaceae, Verbenaceae and Zingiberaceae 10 % each and the rest treated less than 10 %.

### Informant consensus agreement (F_ic_)

This technique is designed to highlight species that have healing potential for specific major purposes. The relative importance of each plant species in treatment of different ailments as categorized in Table 5 was analysed using the Factor Informant Consensus (F_ic_) [41]. F_ic_ values range from 0–1 where values close to one (1) indicate a high rate of informant consensus on a plant species used against an illness category. F_ic_ values close to zero (0) mean low degree of agreement among the informants about the use of a plant species for treatment of a particular ailment. F_ic_ for different ailment categories was calculated to test for homogeneity or consistency of informants’ knowledge about a particular remedy for an ailment category. F_ic_ indicated which plants are widely used and thus merit further pharmacological and phytochemical studies. The highest F_ic_ (0.9) was scored for blood system disorders. The important plants used for anaemia were *Amaranthus dubius* and *Hibiscus acetosella* while those for high blood pressure included *Oxalis corniculata, Canarium schweinfurthi, Sesbania sesban, Vangueria apiculata, Citrus limon* and*, Solanum anguivi.* Seven ailment categories had F_ic_ of zero (0) since each respondent reported a different species used for the same ailment (Table 5).

**Table 5:** Consensus agreement about uses of medicinal plants for ailment categories

Ailment category	N_taxa_	N_ur_	F_ic_
Blood system disorders	11	2	0.9
General conditions	14	9	0.4
Arthritis & Inflammation	29	20	0.3
Infection	52	36	0.3
Neurological & nervous system disorder	16	12	0.3
Sexually Transmitted & venereal diseases	5	4	0.3
Skin infections	69	49	0.3
Gastro intestinal disorders	51	40	0.2
Gynaecological issues	64	50	0.2
Respiratory tract infections	34	27	0.2
Erectile dysfunction, prostate cancer	15	12	0.2
Immune & energy boosting	12	10	0.2
Diabetes	6	5	0.2
Headaches and fatigue	11	10	0.1
Painful body parts	4	4	0
Childcare	10	10	0
Muscular skeletal	9	9	0
Abnormalities	1	1	0
Psychiatric disorders	3	3	0
Body odour	9	9	0
Poisonous animal bites	12	12	0
A taxa may fall in more than one ailment categories			

Key: N_taxa_ - Number of species in each use category

N_ur_ - Number of use reports, F_ic_ - Informant consensus factor

### Fidelity Levels (FL) of preferred plant species

For each of the 10 most preferred plant species a fidelity level (Table 6) was calculated to quantify their importance to treat a major ailment [42]. It was calculated based on the number of users of a given plant species to treat a major ailment. FL shows the proportion in percentage of informants claiming the use of a plant species for the same major ailment to the total number of informants who mention the plant for any use. FL = (I_p_/ I_u_) x 100 where I_p_ = Number of informants who suggested the use of a species for the same major purpose (therapeutic use), (I_u_) = Total number of informants who mentioned the plant species for any use.

**Table 6:** Fidelity Levels (FL) of most commonly used plants by Key Informants

Plant species	Therapeutic uses	I_p_	I_u_	FL%
*Vernonia amygdalina*	Malaria	36	36	100
*Mormodica feotida*	Malaria	31	36	86
*Warburgia ugandensis*	cough	11	28	39
*Prunus africana*	Prostate cancer	3	7	43
*Erythrina abyssinica*	Vomiting	11	11	100
*Piptadeniastrum africana*	Cough	8	9	89
*Albizia coriaria*	Skin infections	8	10	80
*Spathodea campunulata*.	vaginal lubrication	4	8	50
*Mondia whitei*	Erectile dysfunction	6	7	86
*Alstonia boonei*	Prostate cancer	3	4	75

Key: I_p_ - Number of informants who suggested the use of a species for the same major ailment

I_u_ - Total number of informants who mentioned the species for any use

Table 6 shows high fidelity levels of greater than 50 % for seven plant species which highlights the importance of these species in treatment of the mentioned diseases in the study area. *Vernonia amygdalina* and *Erythrina abyssinica* had a fidelity level of 100 % in treatment of malaria and vomiting respectively. High FL levels for these species indicated their outstanding preference for treating malaria and vomiting.

## Discussion

### Characteristics of respondents

Most of the respondents were men with an average age of 52 years. African belief is that traditional healers should be male [43–45]. A high proportion of key informants being male of 50 years and above is in line with studies in Rwanda [46, 47]. Old people (aged 51–80 years) in society have more knowledge on medicinal plants and their uses due to long direct contact with plant resources. In contrast, younger people have little interest in traditional medicine in general and there appears to be a risk of knowledge loss if nothing is done to motivate them. Younger people are exposed to modern education and hence not interested in learning and practicing ethnomedicinal wisdom that would perpetuate indigenous knowledge. Differences in medicinal plants knowledge among age groups was also reported in other studies [48, 49] in Ethiopia.

### Diversity of medicinal plants

The high number of species documented indicates that the study area has diverse flora used in treatment of various ailments and rich traditional knowledge on medicinal plants in the community. This makes Mabira CFR an important source of herbal medicine for the rural communities since more than half of the mentioned medicinal plants were harvested from the forest. High utilisation of medicinal plant species from forests has been reported among the Bakonjo and Bamba in Mt. Rwenzori and Semiliki forest areas in Bundibugyo, Western Uganda [50, 51].

Families Fabaceae, Asteraceae, Euphorbiaceae, Lamiaceae, and Solanaceae are widely reported in herbal preparations in different parts of Uganda [1, 8, 19, 52, 53] and their widespread use could be attributed to their wide range of bioactive compounds. Asteraceae is reported to have a large number of bioactive compounds [54, 55] thus contributing to the high utilization rates of members of the family for medicinal purposes.

A majority of plant species documented treated more than one condition. The use of one plant to treat several ailments is probably attributed to presence of many metabolites in one particular plant and also the fact that the same molecule can be active against different pathogens. In other instances a combination of plants were used in preparation of a herbal remedy against a certain ailment which illustrates the synergistic effects of such plants. As an example *Amaranthus spinosus* and *Cleome gynandra* leaves were used against fungal infections of the scalp, *Balanites aegyptica* roots are mixed with leaves of *Citrus limon* against diarrhoea. On the other hand some remedies were monotherapies based on preparations from a single plant. Such plants could be palatable, nontoxic and highly effective against ailments they are used to treat based on experience of users.

Most of the medicinal plant species collected and identified in the study area were also medically used in other areas of Uganda [1, 19, 56] and other parts of Africa [57] to treat the same or different ailments. The use of the same plant species for similar or different ethnomedicinal uses in different countries is a reliable indication of the bioactivity potential of the documented plant species [58]. Of the 190 medicinal plant species identified in the current study, 34 species were identified earlier in Iganga Eastern Uganda [59], 82 species in Mukono and Mabira forest areas [60], 22 species in Western Uganda [1], 40 species in Mpigi [52] and 30 species in Oyam Northern Uganda [8]. A comparison of ethomedicinal uses of some plant species used in Mabira CFR communities with other parts of Uganda and in other countries is presented in Table 7. Bioactivity studies previously conducted on some of the identified plant species collaborate their ethnobotanical uses. For instance *Capsicum frutescens* is used in management of different cancers – an activity attributed to presence of capsaicin which possesses antimutagenic and anticarcinogenic activities [61]. Also *Prunus africana* has been found to possess anti-inflammatory and antioxidative activities and compounds like cytotoxic phenolics and beta sitostenone, n-docosanol [62] which are important in management of cancer. The ethnomedicinal reports of the same plant species across geographical regions and different cultural groups is indicative of the medicinal properties of the species.

**Table 7:** Relevant literature on previous ethnomedical uses of some medicinal plant species in the current study

Medicinal plant species	Ailments treated in current study	Previous reports of ethnomedical uses	Country of previous use
*Vernonia amygdalina*	Malaria	Malaria	Uganda [63, 101], Ghana [98], Cameroon [102], Democratic Republic of Congo(DRC) [103], Rwanda [104]
		Wounds	Nigeria [105]
		Skin rashes, diarrhoea, herpes zoster, cryptococcal meningitis	Tanzania [106]
		Infertility, amenorrhea	South Africa [107]
		Tonsolitis	Ethiopia [108]
*Ageratum conyzoides*	Uterine pains, helminth infections	Splenomegaly, colic pains, wounds	Uganda [1]
*Vernonia lasiopus*	Malaria, stomachaches	Skin allergy, constipation	Uganda [1]
*Cleome gynandra*	Prolonged labour	Convulsions, diphtheria, toothaches, peptic ulcers, vomiting	Uganda [1, 19]
*Aloe vera*	Malaria	Wounds	Kenya [96]
*Prunus africana*	Enlarged prostate	Prostate and breast cancers, Hypertension	Kenya [96, 109]
*Capscum frutescens*	Prostate cancer	Throat, breast and squamous cell carcinoma	Kenya [109]
*Amaranthus spinosus*	Scalp fungal infections	Haemorrhoids	Nigeria [57]
*Mangifera indica*	Cough, infertility, convulsions	Haemorrhoids	Nigeria [57]

### Plant parts used

The use of leaves to make herbal medicine preparations followed by roots and barks is a common practice in many communities in Uganda as reported in Mukono [60], Sango bay in Southern Uganda [16], Western Uganda [1], communities around Kibale National Park [63], Mpigi [52] and other countries like Kenya [64], Ethiopia [65] and Bolivia [66]. The high utilisation rates of leaves could be attributed to the ease with which they can be obtained in large quantities compared to other plant parts. Leaves are the main photosynthetic organ in plants and considered to be a key component of the natural pharmacy for synthesis of constituents particularly those that are more pharmacologically active against diseases [67]. The preference of leaves to other plant parts is thus thought to be due to accumulation of active ingredients like tannins and other alkaloids [67]. In contrast, in Oyam district of Northern Uganda, roots were the common plant parts used in herbal medicine preparations and the other parts were underutilized [8]. However, as noted [68] a clear relationship exists between the parts of the plant collected, or the collection method and the impact on the harvested plant. Collection of the bark and root is damaging and makes species vulnerable to overexploitation. Harvesting the bark in large quantities can destroy the plant because the protective role of the bark to the plant will be curtailed. On the other hand uprooting plants especially in case of herbs and shrubs causes total destruction of the plant. Debarking and uprooting of medicinal plant species negatively affects the sustainability of the species in use. For species like *Spathodea campanulata, Tamarindus indica* and *Phytolaca dodecandra* in which more than one parts is used; sustainability would probably be achieved if the harvesting of bark and root is avoided and harvesting of leaves which is less destructive is promoted. The use of leaves is less destructive if small quantities are collected but not so if large quantities are harvested. As noted [69], overharvesting of leaves can lead to deterioration of medicinal plants since removal of leaves limits the transformation of vegetative to reproductive development such as flower production and seed/fruit development which in turn limits the natural regeneration of plants. Harvesting of roots on the other hand is more destructive as it often involves uprooting whole plants which consequently affects regeneration for sustainable use.

Herbal preparations made from more than two plant parts of the same plant such as the bark and roots of *Psedospondias microcarpa,* leaves, bark and roots of *Spathodea camapnulata* and the leaves, roots and vines of *Croton macrostachyus* (Table 1) may endanger the species unless mechanisms for sustainable utilisation are put in place. Many studies have showed that leaves of different plants possess bioactive ingredients against different diseases and pathogens [69–72]. Since harvesting of leaves is less destructive than harvesting roots or barks, it is necessary to test leaves for efficacy against different ailments in plants where roots and barks are mostly harvested to minimize dangers of overexploitation. As an example the leaves of *Vernonia amygdalina* have been found to be effective against malaria [73] and thus the harvesting of roots of this species can be avoided.

### Habit of medicinal plant species

Herbs were the most common plant life forms used for medicinal purposes. Harvesting of herbs that are in most cases annual is an indicator that collection of medicinal plants from the forest is not a big threat to conservation. This could be attributed to their abundance throughout the year as reported previously in Uganda [15, 19, 53, 63] although shrubs were reported to be commonly used in northern Uganda [12] and in Ethiopia [74]. The popularity of herbs as a source of herbal therapies is often attributed to their high pharmacologically active ingredients as compared to woody plants [8]. Shrubs are preferred due to their availability all year round since they are relatively draught resistant and are not affected by seasonal variations [65].

### Source of medicinal plants

Traditional healers interviewed lacked medicinal plant gardens and collected medicinal plants from the forest. A similar trend was reported in Zimbabwe [75] but cultivated plants have been used from ancient times such as in Iran and various studies have confirmed potency of chemical constituents in them [14]. However, commercial collectors require large volumes which put pressure on the plant population. Consequently, overexploitation may lead to disappearance of many species of economic value and other uses pausing challenges to their conservation in Uganda’s forests [76] and the African continent as a whole [77].

### Herbal medicine preparation and administration

The main route of herbal medicine administration was oral. This mode of administration is commonly used in many herbal remedies as reported elsewhere [8, 78, 79]. The choice of oral administration may be related to the use of some solvents or additives such as water and food that are commonly believed to serve as a vehicle to transport the remedies. The additives enhance extraction of bioactive molecules during remedy preparation. The additives are also important to minimize discomfort, improve taste and reduce adverse effects such as vomiting and diarrhoea. [80] Decoctions were cited as the most common method of preparation of herbal remedies. Boiling is effective in extracting plant materials and at the same time preserves the herbal remedies for a longer period compared to cold extraction. However, both decoctions and cold extracts do not offer long shelf life for the preparations [81]. As such users continuously harvest medicinal plants which puts them under a lot of pressure that may lead to over exploitation.

### Health conditions treated

Herbal therapies are still preferred in primary health care in Uganda [79] and the world [4]. The use of many herbal remedies for treatment of different ailments has been reported in other studies in Uganda [1, 53] and other countries like India [82] and Ethiopia [65]. Thus the diversity of medicinal plants used meet the varied health care needs of communities of Mabira CFR since many people cannot afford conventional treatment due to wide spread poverty. The high frequency in treatment of gynaecological conditions, digestive disorders and skin infections indicate high prevalence of these ailment categories in the study area. Other ailment categories were not commonly treated implying their low prevalence or limited traditional knowledge in the use of medicinal plants to treat them.

### Informant consensus agreement

Blood system disorders had the highest informant consensus value (F_ic_ =0.9). High F_ic_ values are obtained when only one or a few plant species are reported to be used by a high proportion of informants to treat a particular ailment whereas low F_ic_ values indicate that informants disagree over which plant to use [83]. The high F_ic_ for blood system disorders indicates agreement among respondents on the different plant species used to manage them as well as their significance. Within this category the main condition treated was hypertension (high blood pressure). The prevalence of hypertension was confirmed in a third of adults in Mukono district [84]. The respondents attributed this to age and obesity. A study on screening of bioactive constituents of *Solanum anguivi* fruits which was mentioned as one of the remedies against high blood pressure revealed a lot of bioactive phytochemicals which include alkaloids, flavonoids, tannins, saponins, triterpenoids and phenols. The phenols have the ability to retard lipid oxidation in oils and fatty foods [85] thereby reducing cardiovascular diseases. The low F_ic_ value of zero (0) in the following ailment categories; painful body parts, Childcare, muscular skeletal pains, abnormalities, body odour, psychiatric disorders and poisonous animal bites imply lack of agreement in the plant species used in treatment of such ailments. F_ic_ values close to zero that are indicative of low informant agreement [86] could be attributed to use of same species for many ailments in the community.

### Fidelity level

*Vernonia amygdalina* had a fidelity level of 100 % and ranked highest in the treatment of malaria as had been documented in other parts of Uganda [56, 63]. Its leaf extract has been confirmed for having good anti-malarial effects [87, 88] and through in vitro studies [88, 89]. *Vernonia amygdalina* contains steroid glycosides, sesquiterpene and lactones which are active against *Plasmodium falciparum* [90, 91]. This species has also been found to be clinically effective for the treatment of malaria patients [92]. In human trials, extracts of *Vernonia amygdalina* reduced parastaemia by 32 % [93]. Although *Vernonia amygdalina* is effective for malaria treatment, it can induce labour in pregnant women [1] thus causing miscarriages and therefore should be avoided by them. Species with high fidelity level [94] such as *Vernonia amygdalina* for malaria and *Erythrina abyssinica* for vomiting indicates that these species two were considered of great cultural significance. *Erythrina abyssinica* too has a wide range of use varying from treatment of malaria [95], syphilis [16], tuberculosis [52] to amoebiasis [19] in Uganda. In Kenya *E. abyssinica* is used to treat mumps [96], respiratory tract infections in Mexico [97] and febrile illness in Ethiopia [49]. Its usage for different ailments is possibly due to a wide range of bioactive compounds [95].

Besides malaria, *V. amygdalina* has been used in Uganda to treat various diseases. A decoction from its roots and leaves is used to treat syphilis, ulcers, liver problems [1], its stem bark is used to treat tuberculosis [52] and its roots are used to treat cough, abdominal pain, wounds, hernia and headache [8]. The use of *V. amygdalina* leaves was reported to treat heamorrhoids [57] in Nigeria, malaria [98] in Ghana and in Ethiopia against bloating, dandruff and impotency [49]. The 100 % choice by key informants of using *V. amygdalina* and *E. abyssinca* for treatment of malaria and vomiting is an indicator of the healing potential of these plant species [99]. These results point to the great potential of *V. amygdalina* and *E. abyssinica* for use as sources of new drugs for treatment against malaria and vomiting.

Other species that were preferred in this study were also medicinally important in other areas against the same or different ailments. The use of the same species in different areas against the same ailment confirms the confidence users have in herbal remedies. *Momordica feotida* was used in Uganda to treat sexually transmitted infections and abdominal pain [8], cough [56] and its roots were effective against erectile dysfunction [3]. The stem bark of *Warburgia ugandensis* was effective against tuberculosis in Mpigi while both its roots and bark treated erectile dysfunction in Western Uganda [3]. However, leaves of the same plant were used in Kenya to treat common cold and sore throat [96]. *Alstonia boonei* treated haemorrhoids in Nigeria [57]. The wide spread reporting on the use of these medicinal plants by different communities in different localities could be attributed to different cultural groups which could validate medicinal properties of these species and confirms the confidence users have in the remedies.

The low citation of *Prunus africana* against prostate cancer reflects lack of awareness about the symptoms of the disease, the facts that it is specific to men of a specific age category, the fact that not all men gate prostate cancer and that diagnosis of prostate cancer is not done. It also indicates limited sharing of knowledge about the disease in the study area.

According to [100], plant species with high fidelity level values are considered potential candidates for further pharmacological investigations and deserve priority attention.

Results from computations of F_ic_ and FL do not collaborate each other since they measure different values but also the diseases treated were grouped in categories and no single disease was considered alone in the F_ic_ calculations. This is due to the different formulae used to calculate the two values. FL was calculated based use reports of a plant species to treat an ailment yet F_ic_ was calculated based on consensus among informants for use of plant species to treat different diseases in an ailment category. However, FL values corroborated well with ranking of preferred species.

## Conclusions

The study shows that Mabira CFR habours a wide diversity of plant species used as remedies for several ailments. Such plants are very useful especially to people who cannot afford modern medical care and in cases where access to modern heath facilities is not easy. Knowledge and use of herbal medicine for treatment of various ailments among the local people is still part of their life and culture and this calls for preservation of the integrity of the forest and indigenous knowledge of herbal medicine use. The documented plants have potential of being used in drug development.

### Ethics and approval of the study

Ethical approval of the study was obtained from the Uganda National Council of Science and Technology (UNCST) under registration number SS 3368 after obtaining a research license from National forestry Authority (NFA).

### Consent for publication

Not applicable.

## Abbreviations

CFR: Central Forest Reserve
FL: Fidelity level
F_ic_: Informant Consensus factor
NFA: National Forestry Authority
RRA: Rapid Rural Appraisal
UNCST: Uganda National Council of Science and Technology
